# Annotations

**Published:** 1910-11-19

**Authors:** 


### ANNOTATIONS.

### Facial Asymmetry.

One of the most interesting studies in comparative anatomy is the problem of symmetry. While bilateral symmetry is the rule in both the vegetable and animal kingdoms, there are some notable exceptions to it, of which, so far as plants are concerned, the various species of begonia furnish the best examples. What is usually looked upon as bilateral symmetry, however, turns out on close inspection to be nothing of the kind. Strict bilateral symmetry, so far as the human femora are concerned, for instance, is only met with in 23 per cent. of cases; in the remaining seventy-seven one femur is shorter than the other—a fact, as Sir Frederick Treves has pointed out somewhere, which is consoling to practitioners when called upon to treat a bad fracture of the thigh. Facial asymmetry, again, is equally common, especially in young adults, though it is rarely noticed, except when attention is called to the fact in a photograph or a reflection in a mirror. Most students are aware of the importance, from an examination point of view, of placing a patient with torticollis in front of a looking-glass; but it is not merely in wry neck that facial asymmetry is seen. It is found in many children in whom it is impossible to ascribe a cause to it, and the explanations given are all highly unsatisfactory. That it depends, as some Italian observers have stated, on a relatively inequality of the size of the carotids has now been disproved, and the theories that attitude in sleep, in writing or in standing, or that actual abnormalities in birth are causative factors are admittedly insufficient to explain every case. We know of no exhaustive inquiry into the subject, though many papers have been written on it in connection with combined deformities, such as scarring, torticollis, scoliosis, flat feet, collum varum, and the like. Yet both from an anatomical and a clinical point of view the subject is an interesting one, and should repay investigation. We submit it to the large army of school doctors who are now on the warpath in dealing with the whole and refreshingly wide field of medical inspection of school children. In schools this condition is fairly common, and there are so many factors which may be ascribed as causative that the data collected from such methodical inspection in relation to facial asymmetry ought to be valuable and instructive.

### The Cinematograph in Medicine.

A very large audience assembled on Wednesday, November 9, at the invitation of the authorities of St. Thomas's Hospital, to witness a demonstration by Dr. Levaditi of cinematograph films showing the processes of phagocytosis. The lecturer, who was introduced by Dr. Acland, spoke in French, but Dr. Bashford acted from time to time as interpreter, and thus greatly increased the interest in the lecture for those whose French was deficient. Even apart from the extraordinary interest of the subject, no one could fail to be vividly impressed by the excellence of the films and by the great possibilities of this method of demonstrating pathological and physiological problems. The first series of films were concerned with the behaviour of trypanosomes. The enormous magnification did not entail any sacrifice in clearness; and it is no exaggeration to say that the films were watched with a remarkable intensity even by those to whom the movements of the organisms were already familiar. The contrast in the activities of the trypanosomes in a normal and in an immune serum was exceedingly well brought out. The trypanosomes were seen in the one case to pass close to the leucocyte with impunity, while in the other case, no sooner had a trypanosome approached within the sphere of influence of the white cell than it was irresistibly drawn nearer until contact took place. The subsequent struggling and lashing of cilia, so distinctly shown, could easily be followed until all motion ceased. Films showing clearly the details and movements of spirochætes formed an ample justification of the ultra-microscope. This method of dark background illumination of fresh specimens has made possible the reproduction of these wonderful studies of those hitherto so elusive organisms. Among other very successful films shown were those of agglutination reactions; of hæmolysis, in which the at first boldly outlined red blood corpuscle disappeared completely from view with startling suddenness; of the circulation of the blood in capillaries; and the slow but sure engulfing motion of amœboid corpuscles. Some more "popular" films were afterwards shown, depicting laboratory scenes, such as the inoculation of a mouse with the trypanosome of sleeping-sickness, and the somewhat humorous pictures of the life of a laboratory ape. The projection of many of these films before lay audiences is probably an event shortly to be expected; indeed, we believe already some seven years ago certain films of the circulation of the blood and the typhoid bacillus were shown at the Alhambra. The education of the public mind in this way should do much to destroy the effect of the fabulous tales of vivisection horrors, and to make "understanded of the people" the realities and the enormous value of laboratory work. The lecture was received with enthusiasm by the crowded audience, which included many of the sisters of the hospital and other ladies. Much credit is due to those who at short notice arranged the meeting, and thus afforded so many the opportunity not only of witnessing such unique pictures, but of also hearing one of the most prominent workers in the field of research. At the conclusion of the proceedings a short speech in German by Dr. Bulloch emphasised the polyglot character of the gathering and the great value to medical men of a knowledge of foreign languages, a matter in which, it is to be feared, we still lag behind Continental students. To Pathé Frères is due the credit of the successful production and exhibition of these films.

